# Completeness of individual characteristic data in UK pulmonary rehabilitation

**DOI:** 10.1136/bmjresp-2026-004645

**Published:** 2026-10-26

**Authors:** Holly Drover, Mark Orme, Lin Wang, Matthew Richardson, Jennifer K Quint, Rachael A Evans, Sally J Singh, Enya Daynes

**Affiliations:** 1 Department of Respiratory Sciences, University of Leicester, Leicester, UK; 2 School of Health Sciences, University of Nottingham, Nottingham, UK; 3 Centre for Exercise and Rehabilitation Science, NIHR Leicester Biomedical Research Centre - Respiratory, University Hospitals of Leicester NHS Trust, Leicester, UK; 4 School of Public Health, Imperial College London, London, UK

**Keywords:** Pulmonary Rehabilitation, Pulmonary Disease, Chronic Obstructive

## Abstract

Inequalities in the enrolment and completion of pulmonary rehabilitation (PR) across individual characteristics persist. Accurate data collection of individual characteristics supports services to identify and address these inequalities. This study assessed the completeness of individual characteristic data in a UK National Respiratory Audit Programme dataset of 88 446 individuals assessed March 2019–September 2023. Age, gender and index of multiple deprivation quintile were consistently collected (<1% missing). Ethnicity and Medical Research Council dyspnoea score had substantially higher levels of missing data (14% and 7%, respectively). Improving data completeness may support health inequalities to be identified and addressed in PR services.

 Pulmonary rehabilitation (PR) is a highly effective intervention for individuals with chronic obstructive pulmonary disease (COPD).^1^ However, our recently published study using data from the National Respiratory Audit Programme (NRAP) (England and Wales) reported that younger, female, higher Medical Research Council (MRC) dyspnoea grades and more deprived groups were significantly less likely to enrol in and complete PR.^2^ We also reported that individuals with a Black ethnicity were significantly more likely to enrol in and complete PR than individuals with a White ethnicity.^2^ Using routinely collected healthcare data can support services to identify and monitor inequalities and inform targeted strategies to reduce health disparities.^3^

However, the utility of routinely collected data is reliant on data completeness, as missing data can limit the interpretability of findings. Therefore, this study aimed to assess the completeness of individual characteristic data collected at PR using NRAP data.

NRAP PR workstream continuous data were used. Service users that attended PR with stable COPD and following an acute exacerbation were included. A data access request form was completed and formally approved by the Healthcare Quality Improvement Programme (reference number: 455). All service users included consented for their information to be entered into the national audit and for their data to be shared with third parties with appropriate legal, ethical and security arrangements for research or audit purposes.

The dataset used in this analysis included service users with COPD that attended a PR assessment between 1 March 2019 and 30 September 2023 across 204 services. Data cleaning was conducted by the audit team, and a dataset of 88 446 anonymous service users extracted. Due to dataset anonymisation, it was not possible to reliably identify service users who may have attended a PR assessment and were subsequently recorded in the audit multiple times during the study period.

Individual characteristics collected are age, gender, ethnicity, MRC dyspnoea score and index of multiple deprivation (IMD) (a surrogate measure for deprivation).^4^ To combine the English and Welsh IMD rankings, indices were transformed to quintiles (one - most deprived to five - least deprived). Missing data were summarised using the number and percentage of service users with unrecorded data for each individual characteristic.

Age was consistently collected with no missing data for any service users (table 1). There were minimal missing data for gender (n=89, 0.1%) and IMD quintile (n=701, 0.8%). Ethnicity had the highest proportion of missing data, with 14% (n=12 381) of service users lacking a recorded ethnic group. MRC dyspnoea score was not recorded in 7% (n=6156) of service users. The characteristics of service users with recorded data included in this dataset have been reported in our previously published paper.^2^

**Table 1:** Missing data for individuals with COPD that attended an assessment for pulmonary rehabilitation per characteristic

Characteristic (n=88 446)	Missing data n (%)	Range of missing data across services (%)	Median (IQR)
Age	0 (0.0)	0	0 (0)
Gender	89 (0.1)	0–3.5	0 (0)
Ethnicity	12 381 (14.0)	0–100	4.5 (16.8)
Medical Research Council Dyspnoea score	6156 (7.0)	0–100	2.4 (7.2)
Index of multiple deprivation quintile	701 (0.8)	0–100	0.5 (0.95)

COPD, chronic obstructive pulmonary disease.

While the missing data for age, gender and IMD quintile were minimal, there were substantially higher levels of missing data for ethnicity and MRC dyspnoea score. This may reflect that ethnicity is less consistently recorded in medical records than other individual characteristics, and that MRC dyspnoea score may only be available if specifically assessed during the PR assessment. This is consistent with primary care data, where the missing data for age and sex were minimal, but ethnicity codes were missing in 16.7% of individuals.^5^ Additionally, higher levels of data completeness for some characteristics, such as gender, may be partially attributable to perceived visibility and assumptions made by healthcare professionals.^6^

Patterns of missing ethnicity and MRC dyspnoea score data were explored by comparing the proportion of missing values across PR services. There was considerable variation in the proportion of missing data between services (table 1), potentially reflecting differences in data collection and recording practices across PR services. Systematic under-recording for certain ethnic groups has previously been reported^7^ and may also contribute to the observed patterns. Inaccurate recording or inputting of ethnicity data may lead to selection bias and potentially an inaccurate estimation of the effects of PR. Variation in local data collection process and time pressures in clinical settings have been shown to influence data completeness.^8^ Recent qualitative work reported that PR healthcare professionals felt uncomfortable collecting individual characteristic data, particularly ethnicity, as it was perceived as sensitive information, and felt unable to justify the data collection to service users.^6^ This previous work therefore raises the possibility that missing ethnicity data may disproportionately affect individuals with non-White ethnicities.^7^ This may misrepresent the effectiveness of interventions in non-White ethnicities, limiting further development to ensure inclusivity. Mandated collection of ethnicity data could help ensure that this important data is collected, though it would be essential to provide a ‘prefer not to say’ category to respect service users’ choice. Furthermore, training sessions to support healthcare professionals’ understanding as to the importance of this data and communicating this to service users may also be beneficial.

The proportion of missing data for ethnicity may limit the ability to robustly assess disparities. This is particularly important given health inequalities are well documented in healthcare access, experiences and outcomes across ethnic groups.^9^ Furthermore, national datasets, such as NRAP and other routinely collected datasets including CPRD, include predominantly White individuals.^10^ This, alongside missing ethnicity data, further limits the ability to accurately identify inequalities related to ethnicity. Investing to increase PR enrolment and completion among under-represented groups is expected to provide cost-effective population health benefits.^11^ However, the substantial proportion of missing ethnicity data in studies underpinning these conclusions may limit the generalisability of these findings, as variation in the cost-effectiveness of PR across unreported ethnic groups could influence the overall estimates of cost effectiveness. Missing ethnicity data may also reduce statistical power for analyses of specific ethnic groups, requiring aggregation of ethnic groups to perform powered analyses. Such aggregation risks obscuring important heterogeneity within groups and masking inequalities.

Importantly, we have previously published that missing data groups had significantly lower odds of PR enrolment (MRC dyspnoea score) and completion (MRC dyspnoea score and ethnicity),^2^ indicating that data completeness of individual characteristics could potentially be an indicator of service quality. This is consistent with previous work reporting that completeness of data submitted to national audits can provide an indicator of service quality.^8^ However, it is important to consider that workload, staffing constraints and patient preferences are other factors that may influence incomplete recording.

Additionally, it is concerning that MRC dyspnoea score is unrecorded in 7% of service users, despite an MRC dyspnoea grade of ≥2 being a stated eligibility criterion in the British Thoracic Society Quality Standards.^12^ This finding raises concerns that this important characteristic is not consistently entered into the audit, potentially limiting the ability to accurately assess whether service users meet recommended eligibility criteria.

In conclusion, completeness of individual characteristics collected in the NRAP varied, with ethnicity the least consistently recorded. Without consistent data collection, inequalities across ethnicities and MRC dyspnoea scores within PR services will remain challenging to identify and address. Improving collection and reporting must therefore be a shared responsibility across PR services, NRAP and commissioners if we are to meaningfully improve equity.
